# Phenolic Acids from Fruit By-Products as Therapeutic Agents for Metabolic Syndrome: A Review

**DOI:** 10.3390/ijms26083834

**Published:** 2025-04-18

**Authors:** Ana R. Nunes, Gilberto Alves, Amílcar Falcão, João A. Lopes, Luís R. Silva

**Affiliations:** 1 RISE-Health—Department of Medical Sciences, Faculty of Health Sciences, University of Beira Interior, Av. Infante D. Henrique, 6200-506 Covilhã, Portugal; araqueln@gmail.com (A.R.N.); gilberto@fcsaude.ubi.pt (G.A.); 2 CNC—Centre for Neuroscience and Cell Biology, Faculty of Medicine, University of Coimbra, 3004-504 Coimbra, Portugal; 3 Laboratory of Pharmacology, Faculty of Pharmacy, University of Coimbra, Azinhaga de Santa Comba, 3000-548 Coimbra, Portugal; acfalcao@ff.uc.pt; 4 CIBIT—Coimbra Institute for Biomedical Imaging and Translational Research, University of Coimbra, Azinhaga de Santa Comba, 3000-548 Coimbra, Portugal; 5 iMed.ULisboa, Research Institute for Medicines, Faculdade de Farmácia, University of Lisboa, 1649-003 Lisboa, Portugal; jlopes@ff.ulisboa.pt; 6 CPIRN-UDI/IPG, Center of Potential and Innovation of Natural Resources, Research for Inland Developments (UDI), Polytechnic Institute of Guarda, 6300-559 Guarda, Portugal; 7 CERES-UC—Department of Chemical Engineering, University of Coimbra, 3030-790 Coimbra, Portugal

**Keywords:** by-products, bioactive compounds, metabolic health, valorization, biological properties

## Abstract

The cultivation and processing of fruits generate a wide range of by-products (e.g., pulp, seeds, pomace, leaves, and stems), which are often underutilized despite being rich sources of phenolic compounds with well-documented bioactive properties. The bioactive potential of these compounds has attracted significant interest from both the pharmaceutical and food sectors, offering opportunities for their use in functional foods, dietary supplements, natural medicines, and additives. Among these, phenolic acids have shown promising potential in modulating risk factors associated with metabolic syndrome (MetS), a condition encompassing hypertension, dyslipidemia, hyperglycemia, and abdominal obesity, and contributing significantly to cardiovascular disease. Given the global burden of MetS and the need for novel preventive strategies, numerous studies have investigated the bioactivity of phenolic acids derived from fruit by-products. In this review, we critically examine recent studies regarding the phenolic acid composition of fruit-derived by-products and their biological activity in relation to MetS-related risk factors. This work aims to synthesize current findings, highlight prevailing research trends, and identify existing gaps in the literature to inform future research and promote the sustainable use of fruit by-products in the prevention and management of MetS.

## 1. Introduction

Fruits and vegetables are essential components of a healthy diet due to the numerous nutritional benefits they offer. They are rich sources of vitamins, minerals, fiber, and phytochemicals with antioxidant properties, all of which fundamental to health and well-being [1]. The World Health Organization (WHO) recommends a minimum daily intake of 400 g of fruits and vegetables per day to reduce the risk of chronic non-communicable diseases [2]. While fresh fruit consumption has been widely associated with improved health outcomes, increasing attention has also been directed towards the by-products generated during fruit cultivation and processing [3]. These by-products, such as peels, seeds, pomace, leaves, and stems, are often discarded, leading to environmental and economic challenges [4].

By-products from the cultivation and processing of fruits and vegetables are produced in large quantities. Research indicates that Europe produces around 26,000 tonnes of these bioresidues annually [5]. However, growing evidence indicates that these by-products contain significant amounts of bioactive compounds, including phenolic acids, which have demonstrated antioxidant, anti-inflammatory, antidiabetic, and cardioprotective properties [6,7,8,9]. Notably, compounds such as caffeic acid, *p*-coumaric acid, ferulic acid, and chlorogenic acid are found in high concentrations in citrus fruit peels and sweet cherry stems and leaves [8,10]. These phenolic acids have attracted interest for their potential applications in the food, pharmaceutical, and cosmetic industries, aligning with the principles of the circular economy and sustainable resource use [11].

Metabolic syndrome (MetS), also known as insulin resistance syndrome, is a set of risk factors that can lead to cardiovascular disease (CVD), diabetes, stroke, and other health problems [12]. MetS is diagnosed when a person has three or more of these conditions: hyperglycemia, hypertension, dyslipidemia, low HDL cholesterol, and abdominal fat [13]. Affecting over one billion individuals worldwide, MetS imposes a substantial burden on public health systems and significantly lowers quality of life [14]. In recent years, plant-based compounds, particularly phenolic-rich extracts, have been proposed as adjunctive strategies for the management of MetS and its complications [13,15]. Among these, phenolic acids have shown promise in modulating key metabolic pathways, including glucose regulation, lipid metabolism, and inflammatory signaling [16,17,18].

Despite the increasing number of studies reporting the biological effects of phenolic acids from fruit by-products, this review aims to examine the possible therapeutic role of these compounds in the context of MetS. By integrating findings from in vitro, in vivo, and clinical research, this review seeks to clarify the mechanisms of action of these compounds and identify gaps in current knowledge to guide future investigations.

## 2. Data Collection

The authors of this review conducted a comprehensive and in-depth search for relevant studies on the potential benefits of phenolic acids derived from fruit by-products. Data collection was carried out using various reputable scientific databases, including PubMed, the National Center for Biotechnology Information (NCBI), Google Scholar, Web of Science, Scopus, Science Direct, and other sources of trusted peer-reviewed journals published between 2020 and 2025. However, earlier studies were included to compare and substantiate the findings.

The search strategy involved the use of free terms, keywords, and MeSH terms such as fruits, by-products, phenolic acids, peels, stems, pomace, seeds, phytochemicals, metabolic syndrome, antioxidant activity, anti-hypertensive activity, anti-hyperglycemic activity, and anti-obesity activity. These terms were combined using Boolean operators (AND, OR, and NOT). No restrictions were imposed regarding authorship or publication type during the literature search. It is important to note that this review follows a narrative, rather than systematic, methodology. As such, the process of study selection, while guided by defined keywords and relevance to the topic, was not conducted following a structured protocol (e.g., PRISMA) and is therefore not fully reproducible. This approach allows for flexibility and breadth in the discussion, but it represents a methodological limitation in terms of transparency and replicability.

## 3. Valorization of Fruit By-Products

Since ancient times, fruits, vegetables, or their parts have been used in traditional medicine for the prevention and treatment of many diseases. Recent studies have shown that a diet rich in plant-derived foods can significantly improve health and reduce the risk of developing diseases [8,19]. Fruits are an excellent source of bioactive compounds, including carbohydrates, minerals, phenolic compounds, water-soluble vitamins, amino acids, carotenoids, phytosterols, fibers, and other bioactive substances with health-promoting properties [1]. Furthermore, they have a water content ranging from 70% to 90%, and their seeds have high lipid content, contrary to their pulp and peel. The protein content in fruits is variable and generally low [1].

The global fruit and vegetable industry, while it is rapidly expanding, generates large quantities of by-products during cultivation, processing, and distribution. Common by-products include pomace, peels, pulp, stems, and leaves, many of which are typically discarded, contributing to food waste and environmental burdens [20,21]. According to the Food and Agriculture Organization (FAO), this represents a critical challenge in global food systems, with significant implications for sustainability, food security, and public health [4,21].

**Figure 1:**
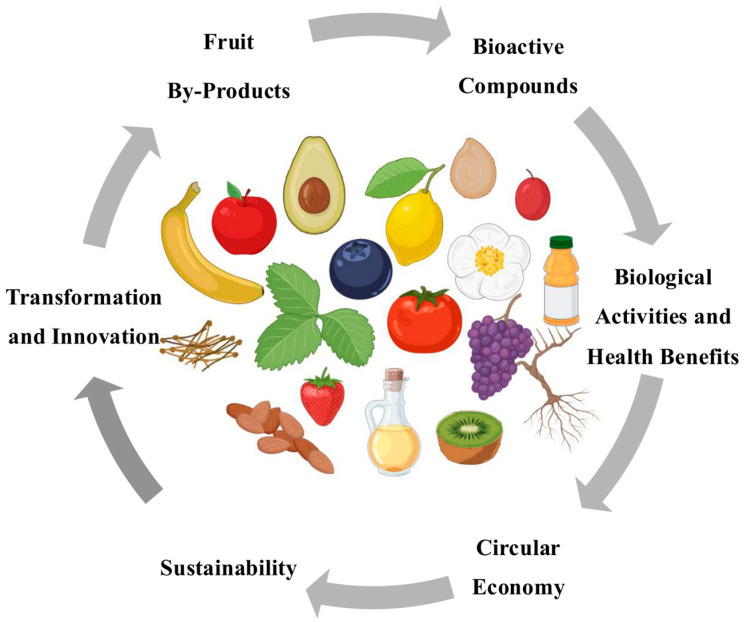
General scheme of the importance of by-product valorization.

The valorization of fruit by-products is a sustainable approach aimed at reusing agricultural waste and turning it into valuable products, thereby reducing its environmental impact and enhancing its economic benefits. Fruit by-products, such as peels, seeds, and pomace, are rich in bioactive compounds, including phenolic acids, flavonoids, carotenoids, and dietary fiber [22]. The efficient utilization of these by-products aligns with the principles of a circular economy and promotes sustainability by minimizing food waste and generating functional ingredients for several industries [23] (Figure 1). Sustainable development seeks to foster and promote this type of economy, aiming to reduce the environmental impact of production processes while maximizing their value. In this context, the search for natural bioactive compounds from renewable raw materials to promote a more balanced, healthier, and more sustainable society has become a global priority. Additionally, an increasing focus on food quality and health promotion has driven consumers to prefer natural ingredients in their food choices [24].

One of the most significant strategies for fruit by-product valorization is the extraction of bioactive compounds, particularly phenolic acids, which exhibit strong antioxidant, anti-inflammatory, and metabolic-regulating properties [8,21,25,26]. Examples include chlorogenic acid in apple peels and pomace [27,28], ferulic acid in citrus peels [29], and gallic acid in grape pomace [30]. The efficient extraction of these bioactive compounds is essential for their practical application. While conventional solvent extraction methods (e.g., ethanol, methanol) are still widely used, emerging green technologies—such as ultrasound-assisted extraction (UAE), enzyme-assisted extraction (EAE), and supercritical fluid extraction (SFE)—offer eco-friendly alternatives with enhanced yield and bioactivity preservation [31].

Given the increasing interest in phenolic acids as dietary and therapeutic agents, a critical evaluation of the evidence surrounding their presence in fruit by-products and their effects on MetS is warranted. The following sections synthesize current knowledge on the biological activity of these compounds, with a focus on their relevance to metabolic health and disease prevention.

## 4. Phenolic Acids

**Figure 2:**
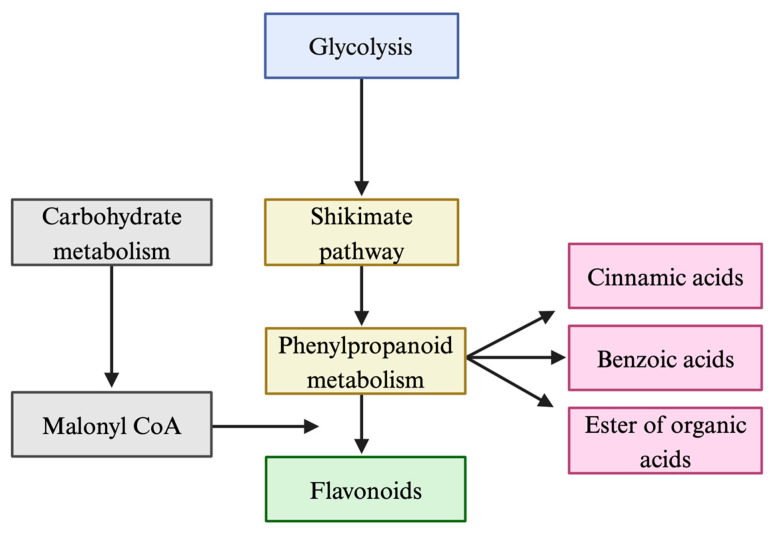
Biosynthesis pathways leading to formation of main groups of phenolic compounds (adapted from Ryan and collaborators [32]).

Phenolic acids are organic compounds widely distributed in nature, found in different sources such as fruits, vegetables, wine, teas, and even their by-products [19]. The biosynthesis of phenolic acids occurs in plants via the shikimic acid pathway, a fundamental process in plant metabolism [32]. Shikimic acid acts as a key intermediate in this metabolic pathway, leading to the formation of a wide variety of phenolic compounds (Figure 2).

Phenolic acids are characterized by the presence of one carboxylic acid group and are generally found in bound forms, such as amides, esters, or glycosides, while their free forms are rarely encountered [33]. Structurally, these compounds have a common base structure consisting of one or more hydroxyl groups (-OH) directly attached to an aromatic ring. The chemical structure of phenolic acids can vary depending on the substituents present on the aromatic ring and the number of hydroxyl groups [34].

**Figure 3:**
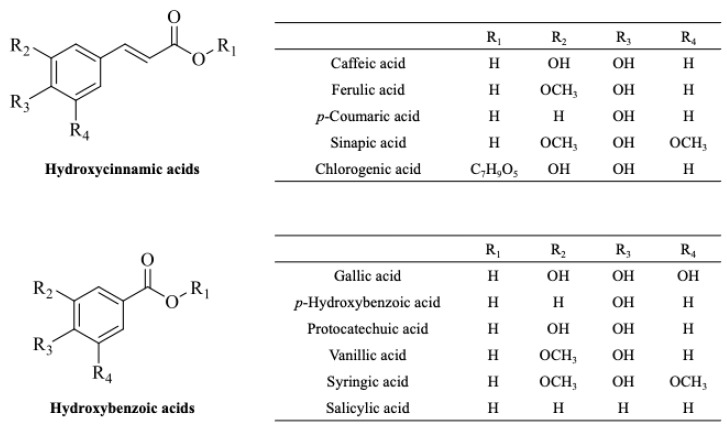
Chemical structures of hydroxycinnamic acids and hydroxybenzoic acids.

Phenolic acids are commonly classified in two main categories: hydroxycinnamic acids and hydroxybenzoic acids [34] (Figure 3). Hydroxycinnamic acids are derivates of cinnamic acid and are often found in foods as simple esters with quinic acid or glucose. For instance, chlorogenic acid is the ester of caffeic and quinic acid [33]. These compounds possess a phenyl ring with at least one hydroxyl group, which contribute to their distinctive properties and wide range of applications [33]. The most common hydroxycinnamic acids include ferulic, caffeic, p-coumaric, and sinapic acids [34]. In contrast, hydroxybenzoic acids are characterized by the presence of a hydroxyl group attached to a benzene ring, typically with a C6-C1 structure. The most common hydroxybenzoic acids include gallic, protocatechuic, ellagic, salicylic, vanillic, and syringic acids [33].

Each type of phenolic acid exhibits specific applications based on its unique biological properties, making them valuable in various industries, including pharmaceuticals, cosmetics, food, and chemicals.

In nature, phenolic acids play an important role in plant resistance against pathogens and environmental stress. These compounds exhibit antimicrobial, antifungal, and antioxidant properties, which help inhibit the growth and reproduction of pathogens and protect plant cells from oxidative damage induced by stress [35,36]. Moreover, phenolic acids present in flowers contribute to their aroma and color, making them more attractive to pollinators. They are also involved in several plant processes, such as cell differentiation, tissue formation, and response to plant hormones. Furthermore, phenolic acids can influence the composition of soil microbiota, thereby affecting nutrient availability for plants and nutrient cycling within the ecosystem [37].

These non-flavonoid compounds have attracted significant interest due to their potential health benefits, particularly their antioxidant, anti-inflammatory, and potentially anticancer properties [33,38]. Upon ingestion, they are absorbed by the gastrointestinal tract and undergo several metabolic pathways in the human body, including methylation, glucuronidation, sulfation, and other reactions that modify their chemical structure [33]. These modifications can influence their biological properties, and the resulting metabolites are primarily excreted through urine and feces [33]. Understanding the metabolic fate of these compounds is crucial, as their extraction from natural sources plays a key role in ensuring their bioavailability and efficacy. The following section explores various techniques used for the extraction of phenolic acids.

### 4.1. Overview of Phenolic Acids Extraction Techniques

The extraction of phenolic compounds is a crucial step for their further utilization in food, pharmaceutical, and nutraceutical applications. Several methods have been developed to enhance yield, efficiency, and sustainability. Since phenolic acids are unevenly distributed in fruits and their by-products due to various factors, their extraction can be challenging [31]. Inadequate extraction methods or single-step procedures may reduce the efficiency of recovering these compounds from plant material. Therefore, selecting an appropriate extraction technique is essential to obtaining the desired phenolic compounds [31]. Below is an overview of conventional and advanced extraction methods for phenolic acids.

#### 4.1.1. Solvent Extraction

Fruits and their by-products contain varying concentrations of phenolic compounds, which can have both simple and complex structures. The interactions of phenolics with other constituents, such as proteins and carbohydrates, represent challenges in selecting an optimal extraction method for all phenolic compounds [39].

Solvent extraction is the most commonly used method for extracting phenolic acids from plant material. This technique relies on the solubility of phenolics in organic solvents such as water, ethanol, methanol, acetone, and ethyl acetate [31]. Phenolic acids dissolve in selected solvents due to their polarity. This method allows for the extraction of phenolics through three distinct approaches: hydro-distillation, maceration, and Soxhlet extraction [40]. The process involves drying and grinding the fruit or by-products, followed by solvent maceration and stirring to dissolve the target compounds. The resulting solution is then filtered, and the solvent is evaporated to concentrate the extract phenolic acids [31].

The solvent extraction method allows for the extraction of 1–30 g of phenolic compounds within 6–24 h. This technique offers advantages such as ease of execution and the flexibility to extract various phenolic compounds using organic solvents with different polarities. Although simple and cost-effective, this method is time-consuming, involves the use of potentially toxic solvents, and lacks selectivity [31]. Key factors influencing these techniques include the type and polarity of the solvents used, their ratio, extraction time and temperature, and the physical and chemical characteristics of the fruits and their by-products [31].

#### 4.1.2. Ultrasound-Assisted Extraction (UAE)

UAE has emerged as a promising method for extracting bioactive compounds from fruits and their by-products and is recognized as a green extraction technology [31]. This method has gained significant attention due to its high efficiency, reduced solvent consumption, and shorter extraction time compared to conventional techniques. Additionally, it is particular suitable for the extraction of thermosensitive compounds [41].

The efficiency of UAE is based on the phenomenon of acoustic cavitation, which occurs when high-intensity ultrasound waves (20–100 kHz) propagate through a liquid medium. This process involves the formation, expansion, and implosion of microscopic cavitation bubbles, generating localized high temperatures and pressures [42]. The resulting microjets and shockwaves induce strong shear forces, breaking down cell walls and enhancing solvent penetration. This increased permeability facilitates the release of phenolic acids into the solvent, improving extraction efficiency [31].

UAE is generally classified into direct and indirect methods. In the direct method, ultrasonic waves are applied directly to the sample–solvent mixture using a sonotrode, an inert acoustic tool submerged in the solution [31]. In the indirect method, ultrasonic waves are transmitted through an ultrasonic bath, allowing multiple samples to be processed simultaneously [31]. Regardless of the method used, an additional purification step is required. Moreover, key parameters such as temperature, sonication, homogenization level, and extraction duration must be carefully controlled [31]. The type and polarity of the solvent also play a crucial role, with ethanol–water and methanol–water mixtures commonly used due to their effectiveness in dissolving phenolic acids while maintaining food-grade safety. Acidified solvents, such as methanol or ethanol with added HCl, are particularly effective in preventing phenolic acid degradation [43].

This extraction method has been successfully applied to several food and agricultural by-products for phenolic acid recovery [43]. It has been extensively used for tracking phenolic acids from citrus peels, apple pomace, grape skins, and coffee husks. In cereal processing, UAE facilitates the recovery of ferulic acid, which is widely utilized in nutraceutical applications, from wheat, rice, and corn bran [44]. Furthermore, it is effective in extracting rosmarinic acid, caffeic acid, and chlorogenic acid from herbs and spices, including rosemary, oregano, and mint [45]. As an emerging green technology, UAE is increasingly being integrated into the food, pharmaceutical, and nutraceutical industries for the sustainable extraction of natural antioxidants.

#### 4.1.3. Microwave-Assisted Extraction (MAE)

Microwave-assisted extraction (MAE) is a modern and effective method for extracting phenolic acids from plant materials. This technique is known for its fast processing, lower solvent usage, and high extraction efficiency, making it a widely adopted approach in food, pharmaceutical, and nutraceutical industries [31]. Similarly to UAE, MAE is particularly beneficial for thermosensitive compounds, as it allows for precise temperature control, minimizing degradation while preserving bioactivity [40].

The principle of MAE relies on the interaction between microwave radiation and polar molecules and ionic compounds within the extraction solvent and plant matrix. Microwaves, typically operating at 2.45 GHz, generate heat through dipole rotation and ionic conduction, which increases the permeability of plant structures, disrupts cell walls, and enhances mass transfer, thereby improving the release of phenolic acids into the solvent [31].

Several factors influence the efficiency of this method, including microwave power and frequency, solvent type, temperature, extraction duration, and the solid-to-liquid ratio. MAE has been effectively applied to a variety of plant sources to extract phenolic acids [46]. In fruits and their by-products, it has been used to obtain caffeic acid, ferulic acid, and chlorogenic acid from citrus peels, apple pomace, and grape skins [47]. In cereal and legumes, MAE efficiently extracts ferulic acid and *p*-coumaric acid from wheat bran, rice husks, and corn residues. Furthermore, in herbs and spices, this technique has proven effective for isolating rosmarinic acid, caffeic acid, and chlorogenic acid [45,46].

MAE is a highly efficient, rapid, and environmentally sustainable method for extracting phenolic acids from plant materials. By optimizing parameters such as microwave power, solvent selection, and extraction temperature, MAE significantly enhances the yield of these valuable bioactive compounds. As a sustainable and emerging green technology, it is increasingly recognized as a viable alternative for the extraction of natural antioxidants in various industrial applications.

#### 4.1.4. Pressurized Liquid Extraction

Pressurized Liquid Extraction (PLE) is an efficient method for extracting phenolic acids. This technique operates by using solvents at elevated temperatures and pressures, maintaining them in a liquid state to enhance the solubility and diffusion of bioactive compounds [48].

PLE offers several advantages over conventional extraction techniques like maceration and Soxhlet extraction, including higher extraction yields, shorter processing times, and lower solvent consumption. The application of high temperatures increases the solubility of phenolic acids, while pressure prevents solvent evaporation, ensuring a more efficient and controlled extraction process. The effectiveness of PLE depends on multiple factors, including solvent type, temperature, pressure, extraction duration, and the physicochemical properties of the plant material [49]. Polar solvents, such as ethanol and water, are commonly used to optimize the extraction of hydrophilic phenolic acids, while less polar solvents, like acetone and methanol, are more suitable for extracting non-polar compounds. By fine-tuning these parameters, PLE allows for the targeted and efficient extraction of phenolic acids, making it a valuable technique in food, pharmaceutical, and nutraceutical applications.

#### 4.1.5. Supercritical Fluid Extraction (SFE)

SFE is an advanced and environmentally sustainable method for extracting phenolic acids. This process utilizes supercritical fluids, primarily CO_2_, which exhibit properties of both liquids and gases, enhancing mass transfer and extraction efficiency [31]. Operating under high pressure and temperature, SFE enables the selective solubilization of target compounds while significantly reducing the need for toxic organic solvents.

The principle of SFE is based on the ability of a fluid to reach its supercritical state when exposed to temperatures and pressures above its critical point. In this state, the fluid gains enhanced diffusivity and solubilization capacity, allowing it to effectively penetrate plant matrices and extract bioactive compounds. The process is highly adjustable, as modifying the pressure and temperature conditions facilitates the selective extraction of different phenolic acids [31].

Several factors influence SFE efficiency, including pressure, temperature, extraction time, and plant material characteristics [50]. Typically, pressures ranging from 100 to 400 bar and temperatures between 40 and 80 °C enhance the solubility and diffusion of phenolic acids. The addition of co-solvents improves the extraction of polar compounds, while smaller particle sizes increase surface area, leading to higher yields. Optimizing extraction time is also crucial to maximize recovery while avoiding the co-extraction of unwanted compounds [51].

Compared to conventional extraction techniques, SFE offers several advantages [50,51]. First, it is an eco-friendly approach that primarily utilizes CO_2_, a non-toxic and recyclable solvent, minimizing environmental impact. Additionally, SFE prevents the degradation of heat-sensitive phenolic compounds due to its moderate operating temperatures. Since CO_2_ is non-polar, co-solvents such as ethanol or water are often added to enhance the extraction of more polar phenolic acids. Another key benefit is that SFE produces extracts free from solvent residues, making it ideal for food, pharmaceutical, and nutraceutical applications [31].

#### 4.1.6. Enzyme-Assisted Extraction (EAE)

EAE is an innovative and eco-friendly technique that use specific enzymes to degrade plant cell wall components, facilitating the release of phenolic compounds into the extraction medium. EAE is considered an efficient alternative to conventional solvent-based methods, offering advantages such as improved extraction yield, reduced processing time, and lower solvent consumption [31].

The mechanism of EAE is based on enzymatic hydrolysis, where enzymes degrade structural polysaccharides, proteins, and other macromolecules that bind phenolic acids within plant tissues [52]. By breaking down these cell wall components, EAE increases the bioavailability of phenolic acids, leading to improved extraction efficiency. Several factors influence the effectiveness of EAE, including enzyme type and concentration, reaction time, temperature, pH, and the characteristics of the plant material. Optimal conditions must be carefully selected to maximize phenolic acid recovery while maintaining enzymatic stability and activity. Typically, mild temperatures (30–60 °C) and pH values suited to the specific enzyme ensure efficient hydrolysis without degrading sensitive phenolic compounds [31].

EAE offers multiple advantages, including enhanced selectivity, which allows for the targeted extraction of specific phenolic acids while minimizing the co-extraction of undesirable compounds. Additionally, it is a sustainable and eco-friendly technology, significantly reducing the reliance on harmful organic solvents and supporting sustainability initiatives in the food, pharmaceutical, and nutraceutical industries [31]. Moreover, this method can be integrated with other extraction techniques, such as UAE and PLE, to further improve efficiency and yield.

This method has been successfully applied to extract phenolic acids from a variety of plant sources, including fruits, vegetables, cereals, and medicinal plants. Current research efforts focus on optimizing enzymatic formulations and process conditions to improve industrial scalability and economic feasibility.

The relevance of the extraction of phenolic acids to health applications, particularly to the management of MetS, underscores their therapeutic potential. By integrating the valorization of fruit by-products with the targeted extraction of bioactive compounds, this approach not only mitigates food waste but also advances the potential for novel treatments for metabolic disorders.

### 4.2. Main Phenolic Acids in Fruit By-Products

Botanically, fruits are reproductive structures that develop from ovary of a flower and serve to protect and facilitate the dispersal of seeds [53]. Depending on the texture of the pericarp, fruits can be classified as fleshy or dry [53]. Fleshy fruits include berries (e.g., grapes, tomatoes) and drupes (e.g., peaches, plums), while dry fruits may be dehiscent or indehiscent, depending on their mechanism of seed release. These classifications, although taxonomic in nature, are relevant when considering the types and distributions of phytochemicals within fruit tissues.

Fruit consumption plays a fundamental role in human nutrition and is associated with the intake of various bioactive compounds, notably phenolics. However, due to their high water content and perishability, large quantities of fruits are lost throughout the supply chain, with estimates of waste reaching up to 30% of the initial mass [4]. The resulting bioresidues, such as peels, seeds, pulp, and leaves are increasingly recognized for their phytochemical richness, particularly in phenolic compounds [54,55]. These compounds have been associated with multiple biological activities, including antioxidant, anti-inflammatory, and antimicrobial effect [19,54].

Among the various classes of phenolic compounds, phenolic acids have attracted particular attention due to their potential therapeutic properties and relatively high abundance in fruit by-products [34]. These compounds are typically divided into two subclasses: hydroxycinnamic acids (e.g., chlorogenic, caffeic, ferulic, and p-coumaric acids) and hydroxybenzoic acids (e.g., gallic, protocatechuic, syringic, and vanillic acids) [56]. Their concentrations and profiles are influenced by several factors, including plant species, geographical origin, maturity stage, and extraction method [56].

The main hydroxycinnamic acids in found in fruit by-products include chlorogenic, caffeic, ferulic, and p-coumaric acids, while protocatechuic, gallic, syringic, and vanillic acids are the main hydroxybenzoic acids found [55] (Table 1). Chlorogenic and caffeic acids are commonly found in by-products of apples, bananas, and lemons. Ferulic acid is primarily present in citrus and cherry by-products, while p-coumaric acid is predominantly detected in grapes [55]. Regarding hydroxybenzoic acids, gallic acid is abundant in the peels of fruits such as pomegranates, grapes, blackberries, and mangoes, and it is also present in grape seeds. Protocatechuic and vanillic acids are frequently found in fruit peels.

While the phytochemical composition data about phenolic acids in fruit by-products are growing, there is a need for a more integrative analysis of their biological relevance, particularly in relation to metabolic health. The following sections of this review explore the current evidence on the health effects of these compounds, with a focus on their mechanisms of action and their potential role in the prevention and management of metabolic syndrome.

**Table 1:** Summary of the main phenolic acids found in fruit by-products.

Fruit	By-Product	Phenolic Acid	References
Apple	Pomace	Protocatechuic acid, chlorogenic acid, 4-hydroxybenzoic acid, caffeic acid, syringic acid, *p*-coumaric acid, ferulic acid, isoferulic acid	[57]
	Peel	Gallic acid, vanillic acid, caffeic acid, chlorogenic acid	[58]
	Pulp	Gallic acid, vanillic acid, chlorogenic acid	[58]
	Seed	Caffeic acid, neochlorogenic acid	[27,28]
Banana	Peel	3,4-Dihydroxybenzoic acid, ferulic acid, chlorogenic acid, gallic acid	[59]
	Inflorescence	Gallic acid, protocatechuic acid, *p*-hydroxybenzoic acid, syringic acid, ferulic acid	[60]
Blueberry	Pomace	Gallic acid, ferulic acid, *p*-coumaric acid, 4-hydroxybenzoic acid	[61,62]
Grape	Juice	Gallic acid, vanillic acid, caffeic acid	[63]
	Pomace	Ferulic acid, *p*-coumaric acid, caffeic acid, vanillic acid, gallic acid, *p*-hydroxybenzoic	[30]
	Seed	Gallic acid	[64]
	Lees	*p*-coumaric acid	[65]
	Peel	Hydroxycinnamic acid derivatives	[65]
Kiwi	Seeds	*p*-coumaric acid, *p*-hydroxybenzoic acid	[66]
	Leaves	Chlorogenic acid, neochlorogenic acid, caffeoylquinic acid	[66]
Lemon	Peel	Caffeic acid, coumaric acid, ferulic acid, sinapic acid	[67]
Olive	Pomace	Gallic acid, vanillic acid, syringic acid, protocatechuic acid, caffeic acid, chlorogenic acid, ferulic acid, sinapic acid	[68]
	Leaves	*p*-hydroxybenzoic acid, vanillic acid, protocatechuic acid, caffeic acid, chlorogenic acid, ferulic acid	[69]
	Seeds	Syringic acid, ferulic acid, caffeic acid derivative	[70]
Orange	Peel	Caffeic acid, *p*-coumaric acid	[10]
	Pulp	Protocatechuic acid, *p*-hydroxybenzoic acid, vanillic acid, caffeic acid, *p*-coumaric acid, ferulic acid, sinapic acid, chlorogenic acid	[71]
Peach	Peels	*p*-coumaric acid, ferulic acid, caffeoylquinic acid, caffeic acid, gallic acid, protocatechuic acid, neochlorogenic acid, *p*-coumaroylquinic acid	[72]
	Seeds	Chlorogenic acid, neochlorogenic acid, gallic acid, caffeic acid, *cis*-5-*p*-coumaroyloquinic acid, *p*-hydroxybenzoic acid	[72]
	Pomace	Chlorogenic acid, neochlorogenic acid	[72]
Pomegranate	Peel	Gallic acid	[73,74]
	Pomace	Gallic acid, *p*-coumaric acid, chlorogenic acid	[75]
Rowanberry	Pomace	Chlorogenic acid	[76,77]
Strawberry	Pomace	Gallic acid, ferulic acid, *p*-coumaric acid, 4-hydroxybenzoic acid	[61]
Sweet cherry	Stems	Caffeoylquinic acid, protocatechuic acid, ferulic acid, hydroxybenzoic acid derivative	[8,78]
	Leaves	Caffeoylquinic acid, *p*-coumaric acid, p-coumaroylquinic acid, protocatechuic acid, ferulic acid	[8,78]
	Flowers	Caffeoylquinic acid	[8,78]
	Pomace	Syringic acid, vanillic acid, chlorogenic acid, 3,5-dicaffeoylquinic acid	[79]
Tomato	Peel	Caffeic acid, vanillic acid, ferulic acid, sinapic acid, chlorogenic acid, gallic acid, *p*-coumaric acid	[80]
	Seeds	Caffeic acid, vanillic acid, ferulic acid, sinapic acid, chlorogenic acid, gallic acid, *p*-coumaric acid	[80]

## 5. Phenolic Acids in the Prevention of Metabolic Syndrome

MetS represents a growing global health burden, characterized by a cluster of risk factors, namely hyperglycemia, hypertension, dyslipidemia, and abdominal obesity, that markedly increase the risk of developing CVD, type 2 diabetes, and other chronic conditions [13,81,82]. The etiology of MetS involves complex interactions between genetic predisposition and environmental factors, particularly dietary habits and physical inactivity [83]. At the molecular level, oxidative stress and chronic low-grade inflammation are central contributors to the pathogenesis and progression of MetS [84].

**Figure 4:**
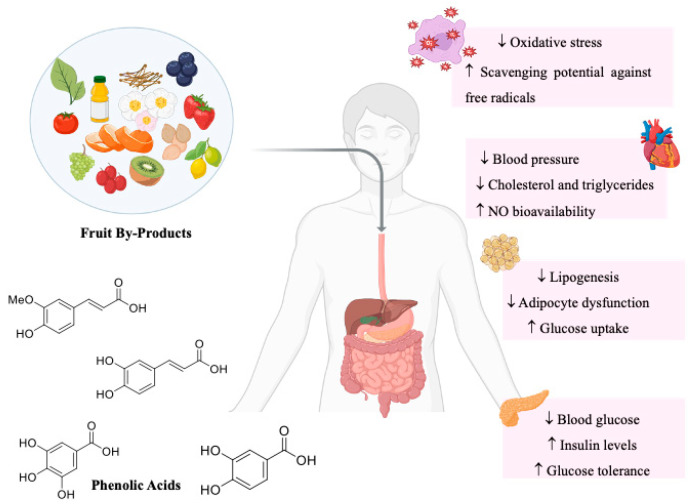
Biological effects of phenolic acids in metabolic syndrome (MetS). (↑—Increase; ↓—Decrease).

In recent years, increasing attention has been given to plant-derived compounds as complementary strategies for the prevention and management of MetS. Among these, phenolic acids have emerged as promising candidates due to their diverse biological activities. Several studies indicate that these compounds exhibit antioxidant, anti-inflammatory, anti-hyperglycemic, anti-hypertensive, and anti-obesity effects [33,34,85]. These properties are particularly relevant given their ability to modulate key metabolic pathways associated with insulin resistance, lipid metabolism, vascular function, and oxidative homeostasis (Figure 4).

Specific bioactivities of phenolic acids are summarized in Table 2, Table 3, Table 4 and Table 5, organized according to their most relevant biological activities: antioxidant (Table 2), anti-hyperglycemic (Table 3), anti-hypertensive (Table 4), and anti-obesity (Table 5). Collectively, current findings suggest that phenolic acids derived from fruit by-products may contribute to integrative strategies targeting the prevention and modulation of MetS.

### 5.1. Antioxidant Activity

Oxidative stress plays a critical role in the pathogenesis of MetS and related non-communicable diseases, primarily though the accumulation of reactive oxygen species (ROS) that promote inflammation, insulin resistance, and vascular dysfunction [4]. Phenolic acids, especially those extracted from fruit by-products, have shown significant antioxidant potential, attributed to their molecular structure, particularly the number and position of hydroxyl groups. These groups contribute to free radical scavenging, metal ion chelation, and the modulation of endogenous antioxidant enzymes [38].

Several studies have demonstrated the antioxidant ability of extracts obtained from industrial fruit by-products. Barbosa and colleagues [86] compared the antioxidant activity of food-grade extracts from apple, lemon, and orange by-products using 2,2-diphenyl-1-picrylhydrazyl radical (DPPH^•^)-scavenging assays. Lemon extract exhibited the highest antioxidant activity (51.7% inhibition), associated with its elevated concentration of hydroxycinnamic acids (407.8 ± 12.17 µg g^−1^), predominantly chlorogenic acid (386.7 ± 11.80 µg g^−1^) [86]. Similarly, Timón and colleagues [87] found that aqueous extracts of lemon, olive, and grape residues showed comparable radical-scavenging effects to commercial rosemary extract.

Mathew and colleagues [88] directly compared the radical-scavenging activity of individual phenolic acids, including hydroxycinnamic acids (e.g., caffeic, ferulic, sinapic, and *p*-coumaric acids) and hydroxybenzoic acids (e.g., gallic, vanillic, and protocatechuic acids), through DPPH^•^, 2,2′-azinobis-3-ethylbenzothiazoline-6-sulfonic acid radical (ABTS^+•^), hydroxyl radical (^•^OH) and superoxide radical (O_2_^•−^)-scavenging assays, as well as a reduction potential assay. The results indicated that hydroxycinnamic acids, particularly caffeic and ferulic acid, exhibited superior DPPH^•^ and O_2_^•−^-scavenging activities, which the authors linked to their higher number of conjugated hydroxyl groups. Among hydroxybenzoic acids, protocatechuic and gallic acids demonstrated significant activity against highly reactive ROS such as ^•^OH [88].

Recent studies have evaluated the antioxidant potential of extracts and isolated compounds from sweet cherry by-products [8,89,90]. Sweet cherry stems and seeds, for example, are rich in neochlorogenic acid and its isomer, both showing strong antioxidant correlations in methanolic extracts [89]. Our research group also demonstrate that hydroethanolic extracts from cherry stems exhibited the strongest DPPH-scavenging activity (IC_50_ = 19.04 ± 0.3 µg mL^−1^), followed by aqueous infusions (IC_50_ = 28.41 ± 0.55 µg mL^−1^), reinforcing the efficiency of solvent-based extraction methods [8]. Bastos and collaborators [91] confirmed similar trends using multiple antioxidant assays, including ferric reducing antioxidant power (FRAP) and thiobarbituric acid-reactive substances (TBARS). All stem preparations showed notable antioxidant activity, with hydromethanolic extracts exhibiting the highest potential. The EC_50_ values ranged from 0.36 to 0.63 mg mL^−1^ for DPPH, from 0.18 to 0.44 mg mL^−1^ for FRAP, from 0.30 to 0.42 mg mL^−1^ for lipid peroxidation, and from 0.07 to 0.24 mg mL^−1^ for TBARS [91]. The literature suggests that hydroxycinnamic acids account for approximately 9.4% of the total phenolic content in hydroethanolic extracts of cherry stems, with 3-*O*-caffeoylquinic acid being the predominant compound [78].

Gonçalves and colleagues [92] assessed the antioxidant activity of major phenolic acids in red fruits and confirmed the strong scavenging activity of caffeic and chlorogenic acids. Notably, their IC_50_ values in DPPH assays were lower than those of ascorbic acid, a known antioxidant standard, suggesting potent radical-neutralizing effects.

Overall, the antioxidant capacity of phenolic acids from fruit by-products underlines their potential as functional ingredients in mitigating oxidative damage related to MetS. Nonetheless, the variability in extraction efficiency, compound bioavailability, and in vivo effectiveness warrants further investigation. Future research should prioritize standardized extraction protocols, comprehensive in vivo studies, and the assessment of synergistic effects between phenolic acids and other phytochemicals.

**Table 2:** Overview of the antioxidant activity of the main phenolic acids found in fruit by-products.

By-Product/Phenolic Acid	Study Type	Main Outcomes	References
	***In vitro* studies**		
Apple, lemon, and orange by-products (unspecified)	DPPH^•^-scavenging activity	Lemon extract presented the highest inhibition of DPPH^•^ (51.7%), followed by apple (39.9%)	[86]
Tomato, grape, lemon, olive and pomegranate by-products		Olive, grape, and lemon pomaces were able to inhibit in vitro oxidation more efficiently than the rest of extracts	[87]
Ferulic acid		Scavenging efficiency was in the following order: Caff > Prot > Gall > Sina >Feru > *p*-Coum > Vani	[88]
Caffeic acid			
Sinapic acid			
*p*-Coumaric acid			
Protocatechuic acid			
Gallic acid			
Vanillic acid			
Sweet cherry petioles		All cultivars exhibited significant antioxidant potential, with DPPH-scavenging values ranging from 29.88% to 86.94%. Moreover, genotypes with the highest phenolic content showed the highest DPPH-radicals scavenging activities	[93]
Sweet cherry stems		Hydromethanolic extract revealed higher antioxidant potential	[91]
Sweet cherry leaves, stems, and flowers		Hydroethanolic stems extract demonstrated the strongest antioxidant activity, followed by aqueous infusions. Moreover, leaves showed better antioxidant activity than flowers	[8]
Ferulic acid	ABTS^+•^-scavenging activity	Scavenging efficiency was in the following order: Gall > Feru > Caff > Prot > *p*-Coum Vanillic acid exhibited low scavenging activity Ferulic acid is more effective than *p*-coumaric acid due to the presence of the OCH_3_ group in position ortho to the hydroxyl group	[88]
Caffeic acid			
Sinapic acid			
*p*-Coumaric acid			
Protocatechuic acid			
Gallic acid			
Vanillic acid			
Ferulic acid	O_2_^•−^-scavenging activity	Scavenging efficiency was in the following order: Gall > Caff > Vani > Prot > Ferul > Sina Compounds that possess more than one hydroxyl group in their aromatic ring (e.g., gallic acid, caffeic acid, and protocatechuic acid) exhibited stronger inhibitory power than monohydroxyl substituents (e.g., p-coumaric acid and ferulic acid)	[88]
Caffeic acid			
Sinapic acid			
*p*-Coumaric acid			
Protocatechuic acid			
Gallic acid			
Vanillic acid			
Ferulic acid	Reducing power	Scavenging efficiency was in the following order: Gall > Caff > Prot > Sina > Ferul > *p*-Coum Gallic acid is very strong reducing agent, owing to the presence of three hydroxyl groups Vanillic acid exhibited very low reducing power	[88]
Caffeic acid			
Sinapic acid			
*p*-Coumaric acid			
Protocatechuic acid			
Gallic acid			
Vanillic acid			
Sweet cherry stems		Hydromethanolic extract revealed higher antioxidant potential	[91]
Sweet cherry stems	*β*-Carotene bleaching inhibition	Hydromethanolic extract, infusion, and decoction revealed higher antioxidant potential	[91]

Abbreviations: Caff—caffeic acid; Prot—protocatechuic acid; Gall—gallic acid; Sina—sinapic acid; Feru—ferulic acid; *p*-Coum—*p*-coumaric acid; Vani—vanillic acid.

#### Mechanisms of Action

Oxidative stress and chronic inflammation are recognized as having a relevant role int the pathophysiology of MetS, contributing to insulin resistance, endothelial dysfunction, and adipose tissue inflammation [94]. Phenolic acids exert antioxidant effects both by directly scavenging ROS and by modulating intracellular signaling pathways involved in the endogenous antioxidant response [38,95]. These activities are primarily attributed to the hydroxyl groups (-OH) on their aromatic rings, which act as hydrogen (H^+^) or electron (e^−^) donors, stabilizing free radicals and preventing lipid peroxidation.

Among the hydroxycinnamic acids, ferulic acid is one of the most studied compounds due to its strong antioxidant capacity [33]. Its strong hydrogen-donating ability enables the effective neutralization of ^•^OH and O_2_^•−^. Additionally, the conjugated structure of ferulic acid facilitates resonance stabilization, which suppresses oxidative chain reactions and lipid peroxidation, processes closely associated with insulin resistance and cardiovascular dysfunction [96]. Importantly, ferulic acid also activates the nuclear factor erythroid 2-related factor 2/antioxidant responsive element (Nrf2/ARE) signaling pathway. This leads to the upregulation of key antioxidant enzymes, such as superoxide dismutase (SOD), catalase (CAT), and glutathione peroxidase (GPx), enhancing the oxidative resilience of metabolic tissues including the liver, pancreas, and adipose tissue [97]. Through these mechanisms, ferulic acid contributes to the protection of pancreatic β-cells, preventing glucotoxicity and maintaining insulin secretion; improved endothelial function via reduced ROS in blood vessels, mitigating hypertension; reduced adipose inflammation, thereby attenuating obesity-related complications; and the preservation of mitochondrial function by decreasing ROS production and enhancing ATP synthesis in insulin-sensitive tissues [98,99].

Likewise, caffeic acid demonstrates potent antioxidant effects, particularly through its O_2_•^−^-scavenging ability. It is considered one of the most effective natural compounds against O_2_•^−^ radicals, thereby reducing the oxidative burden in metabolic organs [100]. In addition to direct antioxidant action, caffeic acid exerts anti-inflammatory effects by inhibiting the nuclear factor kappa (NF-κB) signaling pathway. This inhibition results in decreased expression of pro-inflammatory cytokines, including tumor necrosis factor-alpha (TNF-α) and interleukin-6 (IL-6), both of which are commonly elevated in MetS.

Together, these mechanisms support the therapeutic potential of phenolic acids, particularly ferulic and caffeic acids, in mitigating oxidative stress and inflammation, two central components in the development and progression of MetS.

### 5.2. Anti-Hyperglycemic Activity

Elevated blood glucose levels are a hallmark of MetS, largely attributed to insulin resistance, visceral fat accumulation, dyslipidemia, chronic inflammation, endothelial dysfunction, and oxidative stress [101]. Individuals with MetS typically exhibit impaired insulin responses in adipose tissue, liver, and skeletal muscles, which contribute to hyperglycemia [102]. Visceral fat accumulation exacerbates insulin resistance by secreting adipokines that disrupt glucose homeostasis, while dyslipidemia further compromises insulin sensitivity [103]. On the other hand, oxidative stress and systemic inflammation interfere with cellular insulin signaling, perpetuating metabolic dysfunction [104].

Phenolic acids have gained considerable attention for their anti-hyperglycemic effects, acting through multiple mechanisms [33,101,105,106,107]. A key target involves the inhibition of carbohydrate-hydrolyzing enzymes, specifically α-glucosidase and α-amylase, which delay carbohydrate digestion and glucose absorption, thus reducing postprandial hyperglycemia [108]. Jesus and colleagues [78] evaluated the inhibitory potential of infusions and hydroethanolic extracts from sweet cherry leaves, stems, and flowers. Notably, the stem extracts exhibited the strongest α-glucosidase inhibition, with IC_50_ values of 3.18 ± 0.23 μg/mL for aqueous infusion and 7.67 ± 0.23 μg/mL for hydroethanolic extract [78]. These extracts were rich in phenolic acids, including 3-O-caffeoylquinic acid, 5-O-caffeoylquinic acid, chlorogenic acid, ferulic acid, protocatechuic acid, and various hydroxycinnamic and hydroxybenzoic acid derivatives [8,78].

Chlorogenic acid, a major component of fruit by-products [109], has demonstrated a strong dual inhibition of α-amylase and α-glucosidase in a dose-dependent manner [110,111]. In diabetic mice, chlorogenic acid significantly improves glucose tolerance, reduces insulin resistance, and lowers body weight, with effects comparable to metformin [112]. In human trials, chlorogenic acid has been shown to reduce postprandial glucose and insulin responses during oral glucose tolerance tests, as well as improving fasting glucose levels and insulin sensitivity in individuals with impaired glucose tolerance [113].

Other phenolic acids, such as caffeic, ferulic, gallic, and protocatechuic acids also exhibit strong anti-hyperglycemic properties. Studies in high-fructose-diet-induced MetS rat models show that these compounds restore blood glucose to control levels and normalize key metabolic hormones such as insulin, leptin, and adiponectin [101,114]. Intraperitoneal administration of caffeic acid in alloxan-induced type 1 diabetic mice led to significant glucose reduction and lipid profile improvement [115]. Similarly, ellagic and gallic acids have shown promising effects in lowering blood glucose in various preclinical models [116,117,118,119].

Beyond enzyme inhibition, phenolic acids modulate glucose transport and insulin signaling. These compounds upregulate the expression and activity of glucose transporters (GLUTs), enhancing cellular glucose uptake [33]. Ferulic acid-rich rice bran extracts significantly reduced hyperglycemia and elevated plasma insulin in diabetic C57BL/KsJ-db/db mice [105]. Gallic acid has been shown to enhance GLUT4 translocation and glucose uptake in muscle and adipose cells through mechanisms independent of protein kinase activation [106,108].

Collectively, these findings support the therapeutic potential of phenolic acids in the management of hyperglycemia and highlight the value of fruit by-products as sustainable sources of functional bioactive compounds.

**Table 3:** Overview of the anti-hyperglycemic activity of the main phenolic acids found in fruit by-products.

By-Product/Phenolic Acid	Study Type	Main Outcomes	References
	***In vitro* studies **		
Sweet cherry stems, leaves, and flowers	Enzyme inhibition	Inhibition of *α*-glucosidase enzyme	[78]
Caffeic acid			[120]
Syringic acid			
Chlorogenic acid		Inhibition of *α*-amylase and *α*-glucosidase enzymes	[110,111,121,121]
Gallic acid	Glucose uptake	↑ GLUT4 translocation and glucose uptake in an Akt-independent manner	[106]
	***In vivo* studies**		
Chlorogenic acid	Glucose uptake	↓ Body weight ↑ Glucose tolerance ↑ Insulin sensitivity	[112]
Ellagic acid		Stimulation of glucose-stimulated insulin secretion from isolated islets ↑ Glucose tolerance	[116,122]
Gallic acid		↑ Glucose uptake ↓ Hyperglycemia Improved oral glucose tolerance test Upregulated insulin signaling proteins Enhanced glycogenesis and glycolysis	[107,118]
Caffeic, ferulic, gallic, and protocatechuic acids	Blood glucose	↓ High-fructose-diet-induced metabolic syndrome in body mass index and blood glucose levels	[101]
Caffeic acid		↓ Blood glucose ↑ Insulin levels ↑ Glucose tolerance ↑ Pancreatic β-cell function and morphology	[114,115]
Ellagic acid		↓ Fasting blood glucose ↓ Insulin resistance	[117,122]
Gallic acid		↓ Blood glucose ↑ Insulin levels	[119]
Ferulic acid		↓ Blood glucose ↑ Insulin levels	[105]
	**Human studies**		
Chlorogenic acid	Glucose tolerance	↓ Glucose and insulin concentrations 15 min after an oral glucose tolerance test	[123]

Note: (↑—Increase; ↓—Decrease).

#### Mechanisms of Action

Phenolic acids contribute to the maintenance of glucose homeostasis and improve the body’s response by modulating key enzymes and signaling pathways involved in carbohydrate metabolism [113,114]. A well-documented mechanism involves the inhibition of α-amylase and α-glucosidase, two enzymes responsible for the hydrolysis of complex carbohydrates into absorbable monosaccharides [110]. By suppressing the activity of these enzymes, phenolic acids slow the digestion and absorption of dietary carbohydrates, reducing postprandial hyperglycemia and preventing excessive glucose fluctuations that contribute to insulin resistance. In addition to enzyme inhibition, phenolic acids stimulate the activation of AMP-activated protein kinase (AMPK), a central energy sensor that regulates glucose and lipid metabolism [99]. Once activated, AMPK promotes glucose uptake in insulin-sensitive tissues such as skeletal muscle and liver by increasing GLUT translocation to the plasma membrane [124]. This facilitates cellular glucose entry, resulting in reduced circulating glucose levels and improved insulin sensitivity. Moreover, AMPK activation promotes fatty acid β-oxidation and suppresses hepatic gluconeogenesis, contributing further to systemic metabolic balance [96].

Another key mechanisms is the regulation of GLUT4, which is essential for insulin-mediated glucose uptake into muscle and adipose tissues. Reduced expression or impaired translocation of GLUT4 is a hallmark of insulin resistance in MetS [125]. Several phenolic acids have been shown to upregulate GLUT4 expression and facilitate its translocation, thus enhancing glucose clearance from the bloodstream and restoring insulin responsiveness [126,127].

These mechanisms promote improved glucose utilization, enhanced insulin sensitivity, and greater metabolic efficiency. By simultaneously slowing glucose absorption, enhancing cellular uptake, and regulating energy metabolism, phenolic acids present promising potential as dietary components or adjunctive therapies in the prevention and management of metabolic disorders, including MetS and type 2 diabetes.

### 5.3. Anti-Hypertensive Activity

Hypertension is one of the key factors of MetS, and it is characterized by persistently elevated blood pressure levels, which can lead to severe complications such as a stroke, myocardial infarction, and kidney damage. The pathophysiology of hypertension in MetS is multifactorial, involving mechanisms such as insulin resistance, obesity, endothelial dysfunction, and sympathetic nervous system overactivity [128]. Among these factors, endothelial dysfunction—a hallmark of hypertension—is primarily driven by hyperglycemia and oxidative stress. Elevated blood glucose levels and an excessive production of ROS reduce the production and bioavailability of NO, a crucial vasodilator, leading to impaired vascular function [129,130].

Several studies have demonstrated that phenolic acids can alleviate hypertension by scavenging ROS, regulating NO levels, and mitigating oxidative stress. Their antioxidant properties enable them to neutralize free radicals, chelate pro-oxidant metal ions, and modulate endogenous antioxidant enzyme systems. In particular, chlorogenic acid, caffeic acid, and ferulic acid are known to enhance NO bioavailability, thereby improving endothelial function [33].

Historically, ferulic acid has been used in traditional Chinese medicine for its anti-hypertensive properties [131]. In a study by Suzuki and collaborators [132], ferulic acid was shown to restore endothelial function in the aorta of spontaneously hypertensive rats (SHRs), likely by increasing NO bioavailability. Previously, the same research group reported that a single oral dose of ferulic acid (50 mg Kg^−1^) significantly reduced blood pressure in SHR within one hour, an effect comparable to that of captopril, an angiotensin-1-converting enzyme (ACE) inhibitor [108]. Furthermore, a positive correlation was observed between plasma ferulic acid concentrations and reductions in blood pressure. Long-term administration of ferulic acid also decreased blood pressure in SHRs treated with L-N^G^-Nitro arginine methyl ester (L-NAME), a nitric oxide synthase inhibitor [133]. Similarly, gallic acid was found to suppressed hypertension in both L-NAME-treated mice and SHR models, reinforcing its potential role in modulating vascular function and oxidative balance [134,135]. In another study, a rice bran fraction rich in ferulic acid effectively prevented the development of hypertension in stroke-prone SHRs [136].

In addition, Agunloye and colleagues [137] investigated the effects of caffeic acid and chlorogenic acid on systolic blood pressure, heart rate, and the activity of key enzymes involved in blood pressure regulation, including ACE, acetylcholinesterase (AChE), butyrylcholinesterase (BChE), and arginase, in a model of cyclosporine-induced hypertension [138]. After seven days of treatment, both phenolic acids significantly reduced systolic blood pressure and enzyme activity, improved NO bioavailability, increased catalase activity, and enhanced reduced glutathione content, demonstrating their multifaceted potential in attenuating hypertension through both antioxidant and enzymatic modulation pathways.

**Table 4:** Overview of the anti-hypertensive activity of the main phenolic acids found in fruit by-products.

By-Product/Phenolic Acid	Study Type	Main Outcomes	References
	***In vitro* studies**		
Gallic acid	Human umbilical vein endothelial cells (HUVECs)	↑ NO levels Inhibit angiotensin-I converting enzyme (ACE)	[134]
	***In vivo* Studies**		
Gallic acid	Spontaneously hypertensive rats (SHRs)	↓ Blood pressure	[134]
	N-nitro-L-arginine methyl ester (L-NAME)-induced hypertensive mice	↓ Systolic blood pressure	[135]
Ferulic acid	Normotensive Wistar Kyoto (WKY) rats and spontaneously hypertensive rats (SHRs)	↑ NO bioavailability and ↓ NADPH-dependent superoxide anion levels in SHR aortas ↑Acetylcholine-induced endothelium-dependent vasodilation in SHR	[132]
	Spontaneously hypertensive rats (SHRs)	↓ Systolic blood pressure	[133]
	Male stroke-prone spontaneously hypertensive rats (SHRSP)	↓ Angiotensin-I converting enzyme (ACE) ↓ Blood pressure ↓ Plasma total cholesterol and triglyceride levels	[136]
Caffeic acid	Cyclosporine-induced hypertensive rats	↓ Systolic blood pressure ↓ Cardiac frequency ↓ Angiotensin-I converting enzyme (ACE) ↑ NO bioavailability	[137]

Note: (↑—Increase; ↓—Decrease).

#### Mechanisms of Action

The anti-hypertensive effects of phenolic acids are mediated through multiple mechanisms, including vasodilation, inhibition of the renin–angiotensin system (RAS), reduction in oxidative stress, enhancement of anti-inflammatory activity, and modulation of vascular calcium channels [130]. These actions collectively contribute to improved vascular function and a reduced risk of hypertension in individuals with MetS.

A key mechanism underlying these effects is the enhancement of NO bioavailability [38]. Several phenolic acids, including caffeic acid, ferulic acid, and chlorogenic acid, stimulate endothelial nitric oxide synthase (eNOS), thereby increasing NO production [33]. NO plays a pivotal role in vascular homeostasis by promoting the relaxation of vascular smooth muscle cells, leading to reduced vascular resistance and lower blood pressure levels. In MetS, endothelial dysfunction, characterized by impaired NO synthesis and increased oxidative stress, contributes to hypertension. Phenolic acids help counteract this dysfunction by preserving NO levels and preventing excessive vasoconstriction [138].

In addition to NO-mediated vasodilation, phenolic acids modulate the RAS, a central regulator of blood pressure [139]. The RAS increases vascular tone through the conversion of angiotensin I to angiotensin II, a potent vasoconstrictor. Several phenolic acids, including gallic acid, chlorogenic acid, and protocatechuic acid, have demonstrated inhibitory effects on angiotensin-converting enzyme (ACE), thereby preventing the formation of angiotensin II [119,137]. By reducing vasoconstriction and sodium retention, ACE inhibition results in decreased vascular resistance and blood pressure, underscoring the therapeutic potential of phenolic acids as natural anti-hypertensive agents.

Chronic low-grade inflammation is another key contributor to hypertension in MetS. Elevated levels of pro-inflammatory cytokines such as tumor necrosis factor-alpha (TNF-α), interleukin-6 (IL-6), and interleukin-1*β* (IL-1*β*) promote vascular dysfunction by increasing oxidative stress and impairing endothelial function [81]. Phenolic acids, including caffeic acid, chlorogenic acid, and p-coumaric acid, exert significant anti-inflammatory effects by inhibiting the NF-κB and MAPK signaling pathways, leading to decreased production of pro-inflammatory cytokines [140,141]. Moreover, they downregulate the expression of vascular cell adhesion molecules (VCAM-1 and ICAM-1), which limits immune cell infiltration and vascular inflammation. By mitigating chronic inflammation, phenolic acids help preserve vascular elasticity and support blood pressure control.

Another mechanism through which phenolic acids exert anti-hypertensive effects is the regulation of vascular calcium channels [142]. Calcium ions are essential for vascular smooth-muscle contraction, and elevated intracellular calcium levels contribute to increased vascular tone and hypertension. Phenolic acids such as ferulic acid and chlorogenic acid can inhibit L-type calcium channels, thereby preventing excessive vascular contraction [143]. Additionally, these compounds activate potassium channels, which promote smooth muscle relaxation and vasodilation, further contributing to reduced blood pressure.

Taken together, these findings underscore the multifaceted nature of phenolic acids in blood pressure regulation. Through synergistic mechanisms, enhancing NO production, inhibiting the RAS, reducing oxidative stress, suppressing inflammation, and modulating calcium signaling, phenolic acids significantly contribute to improved vascular function and reduced hypertension risk in individuals affected by metabolic syndrome.

### 5.4. Anti-Obesity Activity

Obesity is considered one of the most significant public health concerns, affecting an increasingly younger population, which results in an increase in health problems associated with metabolic diseases [144]. This condition and MetS are closely linked, with abdominal obesity being a primary risk factor for the development of MetS [145]. Moreover, obesity is a risk factor for other chronic diseases such type 2 diabetes mellitus, CVD, several types of cancer, osteoarthritis, and dementia, contributing to a decline in life quality and expectancy [145].

In response to the adverse effects associated with bariatric surgery and synthetic anti-obesity drugs, the search for natural therapeutic strategies has increased [146]. Among these, phenolic acids have attracted attention due to their potential to mitigate obesity and its related complications. These bioactive compounds exert beneficial effects by inhibiting adipogenesis, stimulating lipolysis, modulating gut microbiota, and reducing inflammation, thus contributing to both the prevention and management of obesity [147,148].

Adipogenesis, the process of adipocyte formation from precursor cells, is regulated by transcription factors such as peroxisome proliferator-activated receptor gamma (PPAR-γ) and CCAAT/enhancer-binding protein alpha (C/EBP-α) [149]. Phenolic acids interfere with adipogenesis by downregulating these factors, thereby reducing lipid accumulation and adipocyte maturation [150]. For instance, *p*-coumaric acid has been shown to inhibit the expression of PPAR-γ and C/EBP-α during the late phase of adipogenesis in 3T3-L1 preadipocytes, without affecting cell viability [115]. This compound also stimulates fatty acid β-oxidation via activation of the AMPK pathway in mature adipocytes [151].

Other phenolic acids, including caffeic acid and vanillic acid, have also demonstrated the ability to suppress adipogenic gene expression [151,152,153]. In a study by Chen and colleagues [154], phenolic acids present in the serum of mice fed a blueberry-enriched diet were found to influence pluripotent stem cells from the bone marrow, suggesting a systemic role in anti-adipogenic activity.

Lipolysis is a metabolic process in which triglycerides stored in adipose tissue are broken down into free fatty acids and glycerol, which can then be used as an energy source. This process is essential for maintaining energy balance and preventing excessive fat accumulation [155]. Phenolic acids can activate AMPK and hormone-sensitive lipase (HSL), both of which promote fat breakdown. However, some phenolic acids may exert an antilipolytic effect under certain conditions. Ren and collaborators demonstrated that p-coumaric acid, benzoic acid, and salicylic acid activated the GPR109A receptor (HM74a/PUMA-G), resulting in suppressed lipolysis in cultured human adipocytes [156]. Interestingly, when these compounds were combined, their inhibitory effect was significantly enhanced. For instance, while individual treatments led to modest lipolysis inhibition (17% for trans-cinnamic acid, 26% for p-coumaric acid, and 3% for benzoic acid), the combined treatment suppressed lipolysis by 53%, suggesting a synergistic effect [156].

In addition, phenolic acids such as chlorogenic acid, gallic acid, and ellagic acid have demonstrated the ability to suppress adipocyte differentiation and lipid accumulation in the 3T3-L1 cell model [151,157,158]. Ko and Ku also observed that apple peel extract, rich in phenolic acids, inhibited lipid accumulation in 3T3-L1 cells [159].

A more recent study revealed that gallic acid and protocatechuic acid significantly improved obesity and insulin resistance in male C57BL/6J mice, effects associated with enhanced energy metabolism and AMPK pathway activation, contributing to reduced adiposity [160]. Conversely, rosmarinic acid, chlorogenic acid, caffeic acid, and gallic acid have shown inhibitory effects on hormone-sensitive lipase (HSL) and pancreatic lipase in a concentration-dependent manner in vitro [158].

There is growing evidence that the gut microbiome plays a crucial role in regulating body weight and obesity [160]. Phenolic acids can reach the colon without being absorbed in the small intestine, where they interact with intestinal microbiota. These compounds can act as prebiotics, inhibiting pathogenic bacteria while promoting the growth of beneficial species, thus exerting systemic metabolic effects [161]. For example, mango peel, which is rich in gallic acid, was shown to promote the growth of *Bifidobacterium* and *Lactobacillus* in in vitro fermentation studies [162].

In the context of lipid digestion, phenolic compounds have been investigated for their ability to inhibit pancreatic lipase, the primary enzyme responsible for dietary fat breakdown [147]. According to Buchholz and colleagues [147], the lipase inhibitory activity of phenolics is influenced by (a) the number and position of hydroxyl groups; (b) esterification patterns; and (c) the degree of polymerization. Hydroxycinnamic acids have shown greater lipase inhibition compared to hydroxybenzoic acids, likely due to their more complex structure and higher polymerization, which enhances enzyme interaction. Among phenolic acids, ferulic and caffeic acids have exhibited the most potent inhibitory activity against pancreatic lipase (IC30: 10 µM) [163]. Similarly, pomegranate peel extracts, rich in gallic and ellagic acids, also demonstrated significant lipase inhibitory effects [164].

These findings underscore the multifactorial role of phenolic acids in obesity management. By modulating adipogenesis, promoting lipolysis, regulating lipid digestion, interacting with the gut microbiota, and enhancing energy metabolism, phenolic acids emerge as promising candidates for the development of natural anti-obesity strategies.

**Table 5:** Overview of the anti-obesity activity of the main phenolic acids found in fruit by-products.

By-Product/Phenolic Acid	Study Type	Main Outcomes	References
	***In vitro* studies**		
*p*-Coumaric acid	3T3-L1 cell model	↓ Adipogenesis during the late phase of MDI-induced differentiation ↑ Fatty acid *β*-oxidation via AMPK pathway in mature adipocytes	[151]
Caffeic acid		↓ Expression of transcription factors PPAR-γ and C/EBP-α with a caffeic acid treatment between 10 and 50 μM in a dose-dependent manner ↓ Intracellular ROS	[152]
Vanillic acid		↓ Expression of transcription factors PPAR-γ and C/EBP-α after 8 days of treatment with 25 μM	[153]
Ellagic acid		Inhibition of lipid accumulation in 3T3-L1 cells	[165]
Apple peel			[159]
Chlorogenic acid		↓ Oxidative stress Inhibition triacylglyceride synthesis	[157]
Gallic acid		↑ Apoptosis in 3T3-L1 preadipocytes, which causes a decrease in cell size and number ↑ PPAR-γ expression, improving insulin resistance for glucose metabolism	[158]
*trans*-Cinnamic acid	Primary subcutaneous human preadipocytes	↓ Adipocyte lipolysis via activation of the nicotinic acid receptor GPR109A (HM74a/PUMA-G)	[156]
*p*-Coumaric acid			
Benzoic acid			
	***In vivo* studies**		
Protocatechuic acid	Male C57BL/6J mice	↓ Body weight and ↓ fat mass of C57BL/6J mice induced by high-fat diet	[166]

Note: (↑—Increase; ↓—Decrease).

#### Mechanisms of Action

Phenolic acids exert beneficial effects on obesity through multiple mechanisms, including an inhibition of adipogenesis, stimulation of lipolysis and fatty acid oxidation, modulation of appetite and energy expenditure, gut microbiota regulation, and a reduction in adipose tissue inflammation [134,135].

Adipogenesis is regulated by key transcription factors, including PPAR-γ and C/EBP-α. Phenolic acids such as chlorogenic acid, caffeic acid, and ferulic acid have been shown to downregulate PPAR-γ and C/EBP-α, thereby suppressing adipocyte differentiation and reducing lipid storage [149, 151]. In addition, phenolic acids inhibit sterol regulatory element-binding protein 1c (SREBP-1c), a key regulator of lipogenesis, further limiting fat accumulation in adipose tissue [167]. These effects help prevent excessive fat storage and the expansion of adipose tissue, a hallmark of obesity.

Beyond their direct effects on lipid metabolism, phenolic acids also play a role in appetite regulation and energy balance [168]. Satiety and hunger are modulated by hormones such as leptin, which signals fullness, and ghrelin, which stimulates hunger. In individuals with obesity, a state of leptin resistance and elevated ghrelin levels is commonly observed, leading to increased caloric intake. Phenolic acids have demonstrated the ability to restore the leptin–ghrelin balance, thereby contributing to appetite suppression and reduced food consumption [145].

Furthermore, certain phenolic acids, including chlorogenic acid, caffeic acid, and protocatechuic acid, have been shown to activate brown adipose tissue (BAT) and stimulate the expression of uncoupling protein-1 (UCP-1) [169]. The thermogenic effect of these compounds supports greater caloric burn, highlighting their potential as natural agents for obesity management [169].

## 6. Conclusions and Future Perspectives

The production and processing of fruits generate substantial amounts of by-products, which represent significant environmental and economic challenges. However, these by-products are rich in phenolic compounds, particularly phenolic acids, that exhibit notable bioactive properties. Increasing evidence highlights the promising role of phenolic acids in the prevention and management of MetS, through their antioxidant, anti-inflammatory, anti-hyperglycemic, anti-hypertensive, and anti-obesity effects.

The incorporation of phenolic acids derived from fruit by-products into the pharmaceutical, nutraceutical, and functional food products represents a valuable opportunity to reduce industrial waste, promote environmental sustainability, and support the circular economy. However, despite promising preclinical and some clinical evidence, several critical knowledge gaps remain. Future research should prioritize the standardization of extraction and purification methods to ensure consistency and reproducibility of bioactive compounds. Moreover, in-depth studies are needed to better understand the bioavailability, metabolism, and pharmacokinetics of phenolic acids both in animals and humans. The long-term safety, efficacy, and optimal dosages of these compounds, particularly in the context of chronic diseases like MetS, also require clarification through well-designed clinical trials. In addition, more attention should be given to the interaction of phenolic acids with other dietary components, their potential synergistic effects, and the influence of gut microbiota on their biological activity. From an industrial perspective, developing scalable, cost-effective, and eco-friendly extraction technologies will be essential to enable the practical application of these compounds at a commercial level.

It is important to acknowledge the limitations of the present review. A major limitation of this review lies in its narrative nature, which inherently limits methodological reproducibility. Although efforts were made to ensure transparency and coherence in the selection and analysis of the literature, the absence of a systematic search protocol prevents the exact replication of this review process. This should be considered when interpreting the findings and their generalizability.

Addressing these research priorities will not only advance our understanding of phenolic acids as therapeutic agents but also unlock the full potential of fruit by-products as sustainable, health-promoting resources. Ultimately, such efforts can contribute meaningfully to improving public health outcomes, promoting food system sustainability, and supporting global strategies for waste valorization and economic resilience.

## Abbreviations

The following abbreviations are used in this manuscript:

-OH	Hydroxyl group
^•^OH	Hydroxyl radical
ABTS^+•^	2,2′-Azinobis-3-ethylbenzothiazoline-6-sulfonic acid radical
ACE	Angiotensin-converting enzyme
AChE	Acetylcholinesterase
AMPK	AMP-activated protein kinase
BChE	Butrylcholinesterase
CAT	Catalase
C/EBP-α	CCAAT/enhancer-binding protein alpha
CVD	Cardiovascular disease
DPPH	2,2-Diphenyl-1-picrylhydrazyl
DPPH^•^	2,2-Diphenyl-1-picrylhydrazyl radical
EAE	Enzyme-assisted extraction
eNOS	Endothelial nitric oxide synthase
FAO	Food and Agriculture Organization
FRAP	Ferric reducing antioxidant power
GLUTs	Glucose transporters
GPx	Glutathione peroxidase
HSL	Hormone-sensitive lipase
IL-6	Interleukin-6
IL-1β	Interleukin-1β
L-NAME	L-N^G^-Nitro arginine methyl ester
MAE	Microwave-assisted extraction
MetS	Metabolic syndrome
NCBI	National Center for Biotechnology Information
NO	Nitric oxide
O_2_^•−^	Superoxide radical
PLE	Pressurized Liquid Extraction
PPAR-γ	Proliferator-activated receptor gamma
RAS	Renin–angiotensin system
ROS	Reactive oxygen species
SFE	Supercritical fluid Extraction
SHR	Spontaneously Hypertensive Rats
SOD	Superoxide Dismutase
SREBP-1c	Sterol Regulatory Element-Binding Protein 1c
TBARS	Thiobarbituric Acid Reactive Substance
TNF-α	Tumor necrosis factor
UAE	Ultrasound-assisted extraction
UCP	Uncoupling Protein-1
WHO	World Health Organization
